# A Molecular Dynamics Study of the Structural and Dynamical Properties of Putative Arsenic Substituted Lipid Bilayers

**DOI:** 10.3390/ijms14047702

**Published:** 2013-04-09

**Authors:** Hui-Hsu Gavin Tsai, Jian-Bin Lee, Jian-Ming Huang, Ratna Juwita

**Affiliations:** 1 Department of Chemistry, National Central University, Jhong-Li City, Tao-Yuan County 32001, Taiwan; E-Mails: 0920bin@gmail.com (J.-B.L.); casuter023@hotmail.com (J.-M.H.); raju_c4c4@yahoo.co.id (R.J.); 2 Department of Chemistry, Brawijaya University, Jl. Veteran, Malang 65145, Indonesia

**Keywords:** arsenic-loving life, arsenated-lipid, lipid bilayers, molecular dynamics

## Abstract

Cell membranes are composed mainly of phospholipids which are in turn, composed of five major chemical elements: carbon, hydrogen, nitrogen, oxygen, and phosphorus. Recent studies have suggested the possibility of sustaining life if the phosphorus is substituted by arsenic. Although this issue is still controversial, it is of interest to investigate the properties of arsenated-lipid bilayers to evaluate this possibility. In this study, we simulated arsenated-lipid, 1-palmitoyl-2-oleoyl-*sn*-glycero-3-arsenocholine (POAC), lipid bilayers using all-atom molecular dynamics to understand basic structural and dynamical properties, in particular, the differences from analogous 1-palmitoyl-2-oleoyl-*sn*-glycero-3-phosphocholine, (POPC) lipid bilayers. Our simulations showed that POAC lipid bilayers have distinct structural and dynamical properties from those of native POPC lipid bilayers. Relative to POPC lipid bilayers, POAC lipid bilayers have a more compact structure with smaller lateral areas and greater order. The compact structure of POAC lipid bilayers is due to the fact that more inter-lipid salt bridges are formed with arsenate-choline compared to the phosphate-choline of POPC lipid bilayers. These inter-lipid salt bridges bind POAC lipids together and also slow down the head group rotation and lateral diffusion of POAC lipids. Thus, it would be anticipated that POAC and POPC lipid bilayers would have different biological implications.

## 1. Introduction

Phosphorus is one of the six most important chemical elements, in addition to carbon, oxygen, nitrogen, hydrogen, and sulfur, which are essential for all forms of life. Phosphorus is a component of adenosine triphosphate (ATP), a principal energy carrier in the cells of organism. The sugar-phosphate chain forms the structural backbone of DNA and RNA. The phosphate group of phospholipids are a major component of cell membranes. In 2011, Wolfe-Simon and co-workers claimed to have discovered a bacterium strain, GFAJ-1 of the Halomonadaceae, isolated from the arsenic-rich waters of Mono Lake, California, that can use arsenic in place of phosphorus to sustain its growth [1]. While arsenic has some chemical similarities with phosphorus, it is usually toxic to most living things. The GFAJ-1 bacteria however had incorporated arsenic into their DNA and proteins and radiolabeled ^73^AsO_4_^3−^ experiments showed a small fraction of ^73^AsO_4_^3−^ was also present in the lipids. This study, however, received many controversial comments. One of the comments is that the arsenate compounds would undergo very rapid hydrolytic cleavage in water [2]. In contrast, the phosphodiester linkages of native DNA is stable with a hydrolysis half-life of approximately 30,000,000 years at 25 °C. Nevertheless, a theoretical functional density study, performed by Gu, Leszczynski, and co-workers [3], revealed that the base-stacking and base-base pairing structure of DNA could increase the stability of arsenate in respect to hydrolysis, in comparison with isolated arsenate model compounds. However, this computational study also suggested that arsenated-DNA is still less stable than normal DNA under hydrolysis.

On the other hand, recent studies revealed that although the bacterial strain GFAJ-1 discovered in California’s Mono Lake is arsenic-tolerant, it requires phosphorus [4]. Further, mass spectrometry did not detect covalently bound arsenated-DNA. Another study, by Erb, Kiefer and co-workers, showed that GFAJ-1 can grow at low phosphate concentrations, even with the co-existence of the high concentrations of arsenate [5]. In contrast, GFAJ-1 cannot grow in a phosphorus-depleted (<0.3 μM) and arsenate-containing environment. The study of Elia,Wellner and co-workers showed that the “arsenic-life” bacteria strongly prefer phosphorus over arsenic [6]. They tested five types of periplasmic phosphate-binding proteins (PBPs) that can uptake phosphate in bacterial cells. Their results showed that all tested PBPs prefer phosphate to arsenate at least 500-fold; in particular, one of the PBPs of GFAJ-1 shows roughly 4500-fold preference for phosphate over arsenate. This work provides a comprehensive understanding of how GFAJ-1 can grow at a high concentration of arsenate.

**Figure 1:**
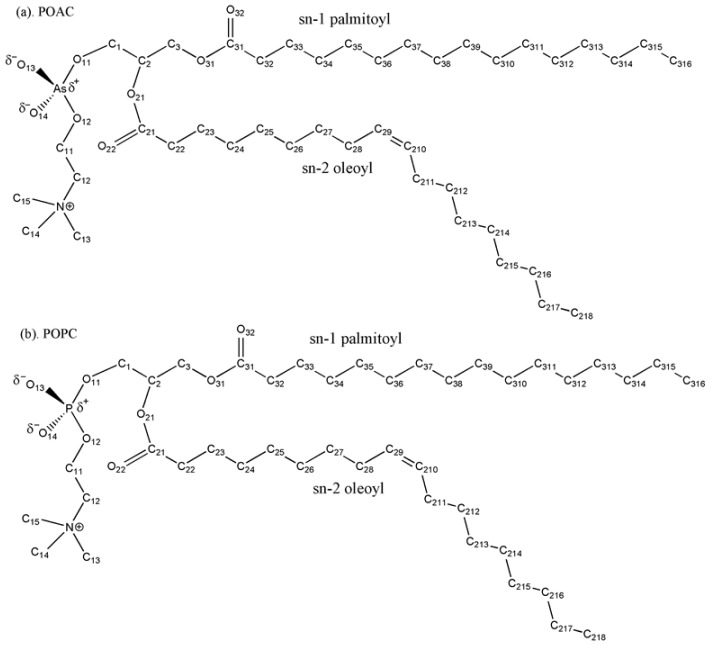
Chemical structures and labeling of atoms of (**a**) POAC and (**b**) POPC lipids.

Although the issue of existence of arsenic-based life is controversial, it remains an open and interesting research topic [7,8] due to the similar chemical properties of phosphorus and arsenic [9]. Arsenate and phosphate have striking chemical similarities. For examples, the arsenate, AsO43-, has the same tetrahedral structure, bonding sites, and nearly identical pKa as phosphate, PO43-. Biological membranes, usually present in the form of phospholipid bilayers, play significant roles in controlling the compartment, transportation, and communication functions of cells [10]. The extensive chemical similarity between arsenate and phosphate raises the question: what are the structural and dynamical differences between putative arsenic substituted lipid bilayers and native phospholipid bilayers? The structural and dynamical properties of lipid bilayers will influence its biological implications. In this study, we studied putative 1-palmitoyl-2-oleoyl-*sn*-glycero-3-arsenocholine (POAC) lipid bilayers in which the phosphorus in 1-palmitoyl-2-oleoyl-*sn*-glycero-3-phosphocholine lipid (POPC) is substituted by arsenic by all-atom molecular dynamics (MD) simulations. The chemical structure of POAC and POPC lipids are displayed in Figure 1. We first developed a force field for the arsenate group in POAC, which is compatible with the CHARMM36 lipid force field. Various structural and dynamical properties of POAC lipid bilayers were calculated; we compared the structural and dynamical properties of POAC lipid bilayers with those of POPC lipid bilayers to understand how the arsenate groups affect the structural and dynamical properties of lipid bilayers.

## 2. Results and Discussion

### 2.1. Area per Lipid and Membrane Thickness

**Figure 2:**
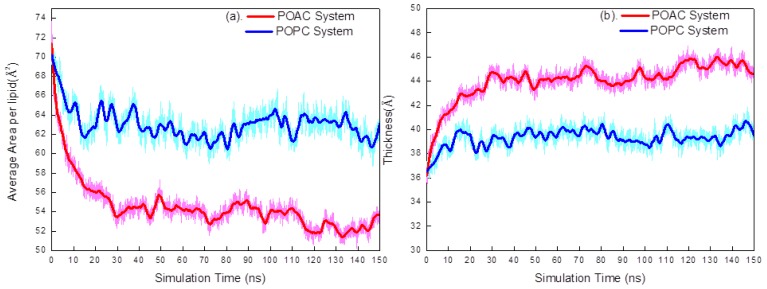
(**a**) The average area per lipid <A> plotted with respect to simulation time for POAC and POPC systems; (**b**) The average membrane thickness <T> plotted with respect to simulation time for POAC and POPC systems. Heavy lines are the average of a 5 ns time window of simulated trajectories.

First, we used the average area per lipid <A> as a parameter to determine the equilibrium configurations of the POAC and POPC simulation systems. The value of <A> was calculated from the lateral surface area of the simulation box divided by the number of lipids in a leaflet. Figure 2a displays the time evolution of the value of <A> for POAC and POPC lipid bilayers. In the POAC systems, the values of <A> decreased rapidly in the first 30 ns, and were stable thereafter. For the POPC simulations, the values of <A> also decreased during the early stage of simulation, and became more stable after approximately 15 ns. Therefore, we used the latter half of the simulation trajectory (75–150 ns) for subsequent statistical analyses. The value of <A> averaged over 75–150 ns trajectories for the POAC system was 53.25 ± 1.13 Å^2^, which is 9.57 Å^2^ smaller than that of the POPC system (62.82 ± 1.22 Å^2^). The experimental values of <A> in POPC bilayers are in a range from 54 to 68.3 Å^2^[11–14]. Our calculated result of <A> is consistent with the experimental values. We also calculated the average membrane thickness <T> in terms of the shortest distance between the phosphorus atom of one lipid in a given leaflet and all of the phosphorus atoms of the lipids in the other leaflet. The values of <T> were averaged over the membrane thickness defined by all of the lipids in a given leaflet. Figure 2b displays the time evolution of the value of <T> for POAC and POPC lipid bilayers, which are stable during the time course 75–150 ns. The value of <T> averaged over 75–150 ns trajectories for the POAC system was 44.79 ± 0.77 Å, which is 5.34 Å thicker than that of the POPC system (39.45 ± 0.65 Å). Our simulated value of <T> in POPC bilayers is in good agreement with the experimental value of 37.0 Å [13]. Poger and Mark have performed delicate studies of structural and dynamical properties of various lipid bilayers [15,16]; they estimated the <A> and <T> values of POPC lipid bilayers are 63.8 Å^2^ and 34.6 Å, respectively. Our estimated <A> and <T> values of POPC lipid bilayers are in good agreement with experimental and simulation results. It is observed that the values of <T> and <A> were generally anti-correlated (Figure 2); this relationship might be understood by considering that when the lipid bilayer lateral area is contracted, its head groups are pushed toward the water phase, resulting in a greater membrane thickness, and *vice versa*.

### 2.2. Atom Distribution

**Figure 3:**
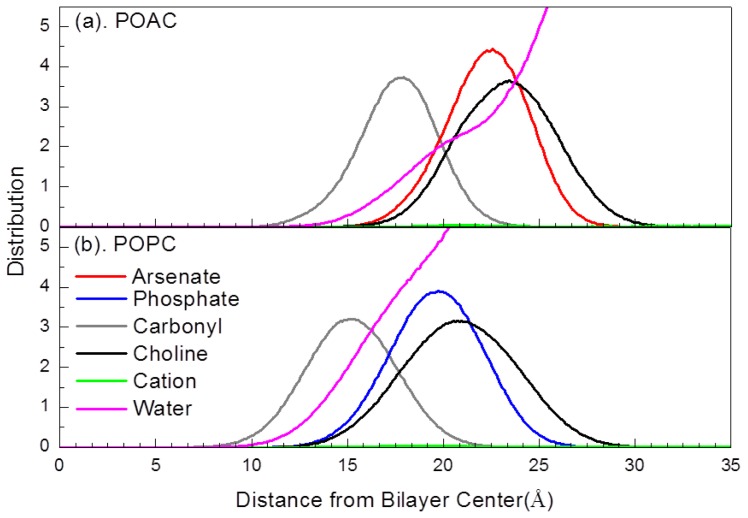
Selected atom distributions of (**a**) POAC lipid bilayers and (**b**) POPC lipid bilayers.

To investigate the structures of POAC and POPC lipid bilayers, we calculated the locations of the lipid head group and carbonyl group atoms (arsenate group contains As, O11, O12, O13, and O14 atoms; phosphate contains P, O11, O12, O13, and O14 atoms; and choline group contains N, C12, O13, C14, and C15 atoms), carbonyl group (C21, O22, C31, and O32 atoms), the ions, and the water molecules from their corresponding lipid bilayers centers (Figure 3). It is observed that the head groups of the POAC system protruded more toward the water phase with respect to those of POPC system. This result is consistent with the calculated membrane thicknesses discussed above: the POAC lipid bilayers were thicker than the POPC system.

**Figure 4:**
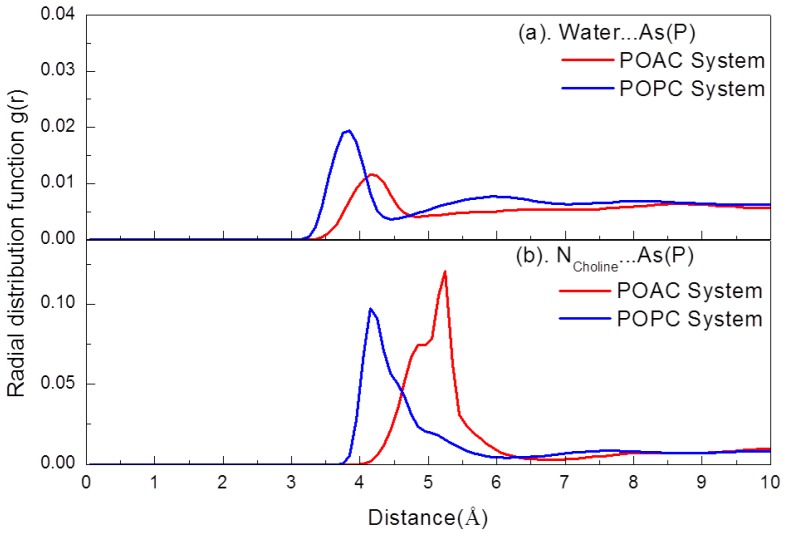
(**a**) Radial distribution functions g(r) of the nitrogen atom of choline groups with respect to water molecules (oxygen atoms); (**b**) Radial distribution functions *g*(*r*) of the nitrogen atom of choline groups with respect to the arsenic atom of POAC lipids and the phosphorus atom of POPC lipids.

In Figure 3, fewer water molecules were associated with the head groups of POAC lipid bilayers relative to that of POPC lipid bilayers. To determine the hydration level of POAC and POPC lipid bilayers, we calculated the radial distribution functions *g*(*r*) for the water molecules (oxygen atoms) with respect to the phosphorus or arsenic atoms (Figure 4a). The *g*(*r*) is calculated by

$$\text{g}(r)=\frac{N(r)}{4\pi r^{2}\rho \delta r}$$

where *N*(*r*) is the number of two chosen atoms at a distance *r*, δ*r* is a spherical shell of thickness at a distance *r* of two chosen atoms, and ρ is the number density. It is observed that the POAC lipids were less hydrated than POPC lipids. The average number of water molecules solvated with one lipid, <H>, is 11.46 for the POAC system, while it is 15.06 for the POPC system. The <H> was calculated with a cutoff distance of 4.85 Å, which covered the first peak of Figure 4a.

To further clarify interactions of inter-lipid arsenate/phosphate-choline groups, we calculated the *g*(*r*) for the nitrogen atoms on choline groups with respect to the phosphorus or arsenic atoms (Figure 4b). The distance of the *g*(*r*) peak for the POAC system is larger than that of POPC system. This result may occur due to the longer bond lengths in arsenate groups than those of the corresponding ones in phosphate groups. The average number of inter-lipid arsenate (phosphate)-choline salt bridges is 4.94 for the POAC system and 3.15 for the corresponding average number of inter-lipid phosphate-choline salt bridge of POPC system; the POAC lipids forms more inter-lipid arsenate-choline salt bridges than the inter-lipid phosphate-choline salt bridge of POPC systems. This result may be due to the higher electrostatic nature of the oxygen atoms of arsenate compared to those of phosphate. The average number of inter-lipid phosphate-choline salt bridges was calculated with a cutoff distance of 6.75 Å, which covered the first peak of Figure 4b.

### 2.3. Order Parameters

**Figure 5:**
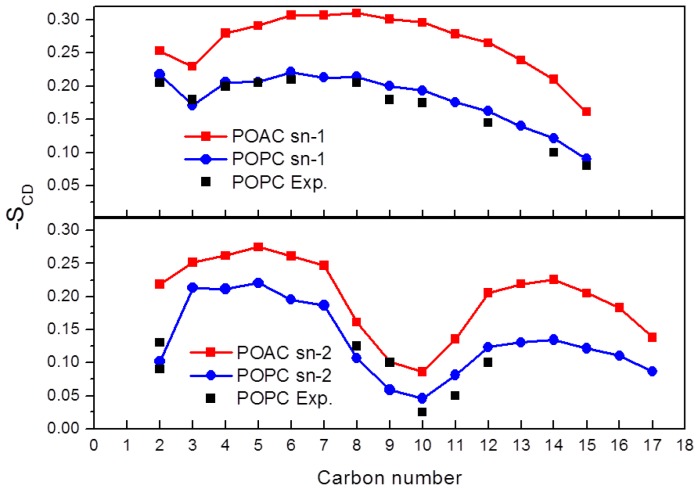
Deuterium order parameters S_CD_ of the (**a**) sn-1 and (**b**) sn-2 chains of POAC and POPC lipid bilayers.

We calculated the deuterium order parameter of the lipid’s acyl chains by the function,

$$S_{\text{CD}}=\frac{1}{2}\langle 3{\text{cos}}^{2}(\theta_{i})-1\rangle$$

where θ*_i_* is the instantaneous angle between the *i*th segmental vector of the carbon atoms of the acyl chain and the normal of lipid bilayers. The symbol 〈 〉 denotes the average over the selected ensembles and simulation time. Figure 5 presents the values of *S*_CD_ of the POAC and POPC systems as well as the experimental values for POPC lipids [17]. For the *sn-1* chain, the atom C31 is labeled as carbon 1, the atom C32 is labeled as carbon 2, *etc.*; for the *sn-2* chain, the atom C21 is labeled as carbon 1, the atom C22 is labeled as carbon 2, *etc.* It is seen that the calculated order parameters of POPC lipids are in good agreement with those of experimental values [17]. For a given lipid bilayer, the *sn-1* chain is more ordered than the *sn-2* chain. Interestingly, the acyl chains of POAC lipid bilayers are more ordered than those of the POPC system. The result was anti-correlated with the values of <A>; it can be understood by that the POAC system with a lower value of <A> would be more compact than the POAC system and, thus, would pack its acyl chains together, resulting in a more-ordered structure.

### 2.4. Dynamics of Lipids

**Figure 6:**
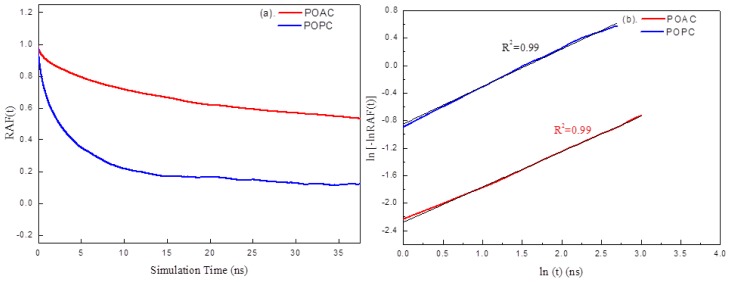
(**a**) RAFs of the POAC and POPC systems and (**b**) Relaxation of the RAF(t) for the POAC and POPC systems.

We studied the head group dynamics by rotational autocorrelation function (RAF) of the P→N or As→N vector to the normal of lipid bilayers (Figure 6a) using the equation.

$$\text{RAF}(t)=<\vec{v_{P\to N}}{\left(t_{o}\right)}_{i}\cdot \vec{v_{P\to N}}{\left(t_{o}+t\right)}_{i}>=\frac{1}{N}\sum_{i=1}^{N}\frac{1}{t_{\text{max}}}\sum_{t_{o}}^{t_{\text{max}}}\vec{v_{P\to N}}{\left(t_{o}\right)}_{i}\cdot \vec{v_{P\to N}}{\left(t_{o}+t\right)}_{i}$$

where *N* is the total number of lipid chains and *t*_max_ is half of the analysis time (to ensure that the RAF results were calculated from the same amount of data). It is observed that the head group rotation of the POAC lipids is slower than that of the POPC lipids. The average angle (in degrees) of the As→N (<θ_As→N_>) vector of POAC lipid head group to the normal of lipid bilayers is 75.6° ± 26.7°, which is larger than that of POPC lipids (66.7° ± 27.3°). To obtain the relaxation time (*τ*) of head group rotation of lipids, the RAF results were fitted to the Kohlrausch-Williams-Watts (KWW) function [18,19],

$$\text{RAF}(t)=exp{\left(-t/\tau \right)}^{\beta }$$

where 0 ≤ β ≤ 1. Generally, it does not fit the early and late regions of the decay very well. The KWW relaxation parameters are determined from the slope and *t* = 1 ns intercept of the ln[−ln RAF(t)] *vs*. ln *t* plot by a least-square fit (Figure 6b). The *τ*_RAF_ for the POAC lipids is 95.02 ns which is approximately 19 times slower than that of POPC lipids (*τ*_RAF_ = 4.99 ns).

We further calculated the lateral diffusion coefficients of lipids in the *xy*-directions (*D_xy_*) by the mean-square-displacement:

$$D_{xy}=\frac{1}{4}\underset{t\to \infty }{\text{lim}}\frac{<{\left[r(t_{o}+t)-r(t_{o})\right]}^{2}>}{t}$$

where *D_xy_* is the lateral self-diffusion coefficient, *r* is the center of mass of the lipid, and *t* is the elapsed time. We obtained that the *D_xy_* for the POAC lipids is 6.75 × 10^−8^ cm^2^/s, which is slower than that of the POPC lipids (*D_xy_* = 9.00 × 10^−8^ cm^2^/s).

Taken together, with respect to the corresponding properties of POPC lipid bilayers, our simulations showed that POAC lipid bilayers have smaller lateral areas, greater thicknesses, and greater order. In addition, the lipids of POAC lipid bilayers have slower head group rotation and lateral diffusion coefficients than those of POPC lipid bilayers. These properties are inter-related and can be understood as follows: when the lipid bilayers’ lateral area is contracted, the head groups of lipids are pushed toward the water phase, resulting in a greater membrane thickness and at the same time, the lipid will have a greater order of their acyl chains within the compact lipid bilayer matrix. In nature, the lipids will have slower motion within a compact lipid bilayer matrix, which retards the lipids’ lateral diffusion and head group rotation. One of the possible reasons for the more compact structure of the POAC lipid bilayers is that more inter-lipid arsenate–choline salt bridges are formed than the corresponding ones of POPC lipid bilayers. These kinds of inter-lipid salt bridges will bind lipids together and retard lipid motion.

The formation of inter-lipid salt bridges may provide additional stabilization, retarding the POAC lipids from being quickly hydrolyzed when they are embedded in their lipid matrix. First, they reduced the water molecules solvated with the arsenate group of POAC lipid. We estimated the average number of water molecules solvated with one lipid is 15.06 for the POPC system, while it is only 11.46 for the POAC system. Second, the inter-lipid salt bridges between POAC lipids may raise the activation energy of POAC hydrolysis by providing a “heavy” leaving group; this effect is similar to the base-stacking in DNA which increases the resistance of As–DNA hydrolysis [3]. The hydrolysis stabilization of POAC lipid bilayers should be higher than that of POAC lipid in its free form.

The POAC lipid bilayers have distinct structural and dynamics properties that differ from those of POPC lipid bilayers. Therefore, it would be anticipated that POAC lipid bilayers will have different biological implications from native POPC lipid bilayers. However, a study of the biological properties of POAC lipid bilayers is beyond the scope of this work. Nevertheless, since POAC lipid bilayers have a more compact structure, we might anticipate that POAC lipid bilayers will create a higher energy barrier for inter-cellular chemical exchange and protein transportation than that of POPC lipid bilayers.

## 3. Computational Methods

### 3.1. Arsenate Force Field Parameterization

**Figure 7:**
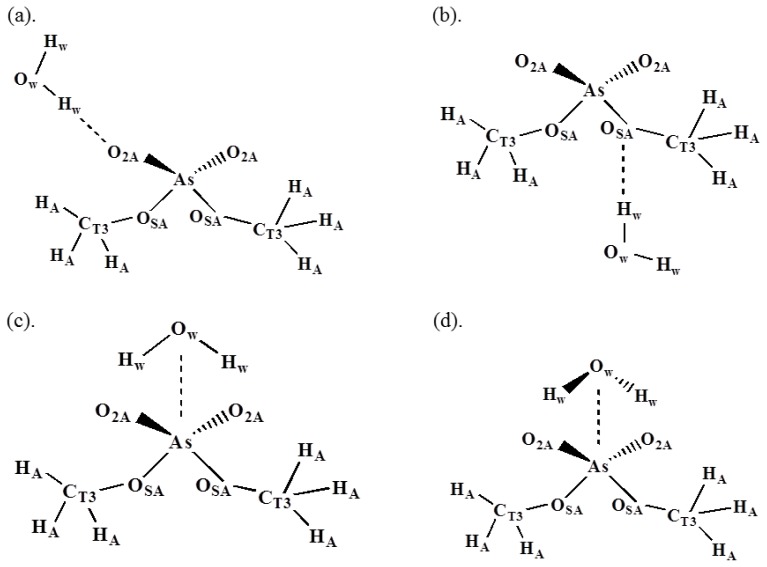
Water-DMA interaction configurations and atom types.

Parameterization of the arsenate group of POAC lipids used the model compounds dimethyl arsenate (DMA, see Figure 7). Parameters of DMA were first developed and then transferred to the dimethyl arsenate group in POAC. To be compatible with the corresponding force fields of POPC, we mainly followed the original methodologies of Karplus *et al.*[20] in developing the parameters of dimethyl phosphate (DMP). All *ab initio* calculations were performed by Gaussian 09 program [21]. The structure of DMA was optimized in the gauche-gauche (two O2A-As-OSA-CT3 dihedral angles) conformation at the HF/6-31G* level. The optimized structures of DMA were used to develop a set of 36 internal coordinates and to construct the B matrix, allowing conversion from Cartesian to internal coordinates. The vibrational spectra of DMA was calculated at the HF/6-31G* level based on its optimized structure. A scaling factor 0.9 was applied to scale the vibrational frequencies of DMA. The U matrix, which permits analysis of the harmonic vibrational frequencies in terms of the potential energy distribution among symmetry-adapted coordinates, was calculated by MOLVIB module implemented in CHARMM program [22].

Molecular mechanics were performed using the CHARMM program for which the potential functions were pre-defined. The energy function includes internal and external non-bonded terms; the internal terms include bond stretching, angle bending, and dihedral angle torsion; the external non-bonded interactions are represented by a Lennard-Jones 6–12 term for the van der waals repulsion and dispersion interaction and a Coulomb term for the charge-charge interactions. Their potential functions are expressed as:

$$\text{U}(\vec{\text{R}})=\sum_{\text{bonds}}{\text{K}}_{\text{b}}{\left(\text{b}-{\text{b}}_{0}\right)}^{2}+\sum_{\text{angles}}{\text{K}}_{\theta }{\left(\theta -\theta_{0}\right)}^{2}+\sum_{\text{dihedrals}}{\text{K}}_{\varphi }(1+\text{cos}(\text{n}\varphi -\delta ))+\sum_{\text{nonbond}}ɛ\left[{\left(\frac{\sigma_{\text{ij}}}{r_{\text{ij}}}\right)}^{12}-2{\left(\frac{\sigma_{\text{ij}}}{{\text{r}}_{\text{ij}}}\right)}^{6}\right]+\frac{{\text{q}}_{\text{i}}{\text{q}}_{\text{j}}}{4\pi {\text{er}}_{\text{ij}}}$$

where *K_b_*, *K*_θ_, and *K*_φ_ are the force constants of bond stretching, angle bending, and dihedral angle torsion, respectively; *b*, *θ*, and φ are the bond length, bond angle, and dihedral angle, respectively; *b_o_* and θ*_o_* are the reference bond length and bond angle, respectively. ɛ is the Lennard-Jones well-depth and σ is the distance at Lennard-Jones minimum, *q_i_* is the partial atomic charge and *e* is the dielectric constant which can depend on *r_ij_* and *r_ij_* is the distance between atoms *i* and *j*.

Non-bonded interaction parameters (point charges, ɛ, and σ_ij_) were optimized to maintain a proper balance between solvent-solute interactions of all configurations shown in Figure 7. The solvent-solute interaction energies were calculated from *ab initio* calculations with the corrections of basis set superposition error [23]. The optimized interaction energy and distance are shown in Table 1, which are good agreement with the HF/6-31G* values for all configurations. The optimized non-bonded parameters are provided in the Table S1 (supporting information).

The empirical potentials of stretching and bending motions were fitted into their corresponding potential energy surfaces of HF/6-31G* calculations. Furthermore, the force constants were re-optimized to the harmonic vibrational frequencies of scaled HF/6-31G* results and normal mode assignments (Table S2) using MOLVIB. The optimized stretching and bending parameters are provided in the Table S1. The next optimization focuses on the torsion parameters. The parameters of three torsion angles, O_2A_-As-O_SA_-C_T3_, O_SA_-As-O_SA_-C_T3_, and As-O_SA_-C_T3_-H_A_, were optimized to fit the adiabatic torsional potential energy surface of HF/6-31G* results. Their torsional potential energy surfaces of empirical calculations and HF/6-31G* calculations were shown in Figure S1 (SI) and they are good agreement with each other. The optimized empirical data of these torsion angles are listed in Table S1 (SI).

### 3.2. Molecular Dynamics Simulations

We simulated the POAC and POPC lipid bilayers by molecular dynamics. The initial configurations of the POAC and POPC systems were prepared using the CHARMM-GUI developed by Im and co-workers [24]. The empirical parameters of the DMA developed above were used to model the arsenate group of POAC lipids, while other groups as well as counter ions were modeled using the CHARMM36 all-atom force field [25]. Water molecules were modeled using the TIP3 model [26]. Each lipid bilayer system consisted of 96 lipids (48 lipids for each leaflet), 4863 water molecules, 13 Na^+^ and 13 Cl^−^ (0.15 M NaCl); the corresponding water–lipid ratio was 50.63. All MD simulations were performed using parallel NAMD 2.7b3 software [27] with an NPT ensemble under three-dimensional periodic boundary conditions. The simulation temperature was controlled to 300 K through Langevin dynamics. The pressure was controlled at 1 bar using the Langevin piston Nosé–Hoover method [28] and the three orthogonal dimensions of the periodic box were allowed to vary independently. The van der Waals interactions were switched smoothly from 9.0 to 12.0 Å to maintain energy stability. Cutoffs of 12.0 and 13.5 Å were used to calculate the pair-wise interactions and generate the list of pairs, respectively. The non-bonded neighboring list was updated every 10 time steps. The particle-mesh Ewald technique [29] was used to treat the long-range electrostatic interactions. The hydrogen atom involved covalent bond lengths were constrained by the SHAKE algorithm [30], allowing the use of a larger integration time step of 2 fs. Prior to MD production runs, conjugate gradient energy minimization was performed to remove the bad contacts of the initial configuration. Next, a 0.1-ns slow heating simulation was performed. The molecular coordinates were stored every 5 ps. For all systems, a 150-ns MD simulation was performed. The trajectories of the final 75 ns simulation were used for statistical analysis. All analyses were performed by our in-house program, Pine-MD, which had been employed in previous MD studies of lipid bilayers [31], amyloidogenic peptides [32,33], and antimicrobial peptides [34]. Several structural and dynamics properties of lipid bilayers were calculated. The analysis methods are described in the Results and Discussion section.

## 4. Conclusions and Summary

Due to the chemical similarities of phosphorus and arsenic, the possibility of sustaining life if the phosphorus is substituted by arsenic has been suggested [1]. We simulated putative POAC lipid bilayers by all-atom molecular dynamics to understand its basic structural and dynamical properties. The POAC lipid is a putative lipid with the phosphorus of POPC lipid substituted by arsenic. We focus on understanding of the structural and dynamical properties of POAC lipid bilayers different from those of its analogous POPC lipid bilayers. Our simulations showed that the structural and dynamical properties of POAC lipid bilayers are distinct different from those of native POPC lipid bilayers. The POAC lipid bilayers have smaller lateral areas, greater thicknesses, and greater order than those of POPC lipid bilayers. Namely, the POPC lipid bilayers are more compact than that of POPC lipid bilayers. The compact structure of POAC lipid bilayers is due to the fact that POAC lipid bilayers form more inter-lipid arsenate–choline salt bridges than that of POPC lipid bilayers, which bind lipids together. Furthermore, these strong inter-lipid arsenate–choline interactions in POAC lipid bilayers also slow down the lipids’ lateral diffusion and head group rotation. Since the POAC and POPC lipid bilayers have distinct structure and dynamics properties, it would be anticipated that POAC lipid bilayers will have different biological implications from native POPC lipid bilayers, e.g., the energy required for inter-cellular chemicals exchange.

## Supplementary Information



## Figures and Tables

**Table 1:** Empirical and HF/6-31G* water interaction energies and geometries for DMA a.

	HF/6-31G*		Empirical b	
		
Interaction	*E_min_*	*R_min_*	*E_min_*	*R_min_*
(a) O_2A_–H_w_	−11.4	1.87	−11.64	1.88
(b) O_SA_–H_w_	−4.77	2.35	−5.03	2.34
(c) As–O_w_^c^	−7.76	4.05	8.24	3.86
(d) As–O_w_^d^	−13.29	3.48	−12.83	3.50

a Energies in kcal/mol and distances in Å. *R_min_* is the water-DMA distance indicated by the dashed line shown in Figure 7;

b Empirical values were obtained from using the CHARMM36 optimized gas phase geometry;

c Interaction (c) has the plane of the water molecule perpendicular to plane of the O=As=O atoms;

d Interaction (d) has the plane of the water molecule in the same plane as the O=As=O atoms.
