# Supplementary material for: A Molecular Dynamics Study of the Structural and Dynamical Properties of Putative Arsenic Substituted Lipid Bilayers
Source: Int J Mol Sci. 2013 Apr 9;14(4):7702–15. doi: 10.3390/ijms14047702 (PMC3645711; doi:10.3390/ijms14047702)
Supplement: Supplementary file 1 [file ijms-14-07702-s001.pdf]

Supplementary Information

Table S1. Parameters for DMA.

**Nonbonded Parameters**			
	q	**ε**	**σ**
As	1.800	−0.785	2.25
O_{2A}	−0.875	−0.135	1.95
O_{SA}	−0.625	−0.195	2.05
**Stretching Parameters**			
	K_b **(kcal/mole-Å^2)**	b_0 **(Å)**	
O_{2A}-As	500	1.61	
O_{SA}-As	250	1.74	
O_{SA}-C_{T3}	330	1.40	
**Bending Parameters**			
	K_θ **(kcal/mole-rad^2)**	θ_0 **(degree)**	
O_{2A}-As-O_{2A}	70	126.5	
O_{SA}-As-O_{2A}	65	106.5	
O_{SA}-As-O_{SA}	55	98.0	
As-O_{SA}-C_{T3}	20	117.4	
O_{SA}-C_{T3}-H_A	60	109.5	
**Torsion Parameters**			
	K_Φ **(kcal/mol)**	***n***	**δ**
O_{SA}-A_S-O_{SA}-C_{T3}	0.55	1	90
	0.85	2	0
	0.60	3	0
O_{2A}-A_S-O_{SA}-C_{T3}	0.10	3	0
X-C_{T3}-O_{SA}-X	−0.13	3	0

Table S2. Vibration frequencies and potential energy distributions from the empirical and scaled HF/6-31G* calculations for DMA [a].

**Scaled HF/6-31G ***					**Empirical**				
**Freq.**	**Assignment %**				**Freq.**	**Assignment %**			
70	t(O–C)	91			74	t(O–C)	86	t(As–H)	15
76	t(O–C)	56	t(As-H)	42	88	t(O–C)	53	t(As–H)	43
98	t(As–H)	89			140	t(As–H)	75	t(O–C)	12
						βO–As–O	11		
132	t(As–H)	55	t(O-C)	41	189	t(As–H)	57	t(O–C)	43
186	twAs–O	48	βAs-O-C	42	233	twAs–O	48	βAs–O–C	29
						βO–As–O	15		
190	βAs–O–C	57	rAs-O	41	235	rAs–O	51	βAs–O–C	35
						wAs–O	13		
281	βO–As–O	45	βO=As=O	42	292	βO–As–O	73	twAs–O	24
310	twAs–O	43	βAs-O-C	38	293	wAs–O	82		
339	wAs–O	82			358	βO=As=O	69	βAs–O–C	23
370	βO=As=O	48	βO-As-O	34	384	βAs–O–C	51	βO=As=O	28
	βAs–O–C	12				twAs–O	20		

Table S2. *Cont.*

Scaled HF/6-31G *					Empirical				
**Freq.**	**Assignment %**				**Freq.**	**Assignment %**			
394	rAs–O	47	βAs–O–C	28	394	βAs–O–C	60	rAs–O	37
	wAs–O	15							
610	vO–As	87			617	vO-As	88		
622	vO–As	87			645	vO-As	88		
901	vO=As	99			903	vO=As	96		
985	vO=As	99			1003	vO=As	97		
1088	vC–O	96			1019	vC-O	81		
1098	vC–O	94			1017	vC-O	81		
1160	rCH_3	95			1137	rCH_3	75	δCH_3	17
1162	rCH_3	94			1137	rCH_3	75	δCH_3	17
1180	rCH_3	90			1153	rCH_3	71	δCH_3	20
1180	rCH_3	91			1156	rCH_3	70	δCH_3	21
1450	δCH_3	96			1622	δCH_3	90		
1451	δCH_3	94			1623	δCH_3	90		
1473	δCH_3	92			1425	δCH_3	78	rCH_3	21
1474	δCH_3	90			1426	δCH_3	78	rCH_3	21
1487	δCH_3	91			1475	δCH_3	76	rCH_3	28
1489	δCH_3	90			1476	δCH_3	75	rCH_3	28
2838	vCH_3	93			2854	vCH_3	100		
2840	vCH_3	94			2854	vCH_3	100		
2880	vCH_3	100			2912	vCH_3	100		
2880	vCH_3	100			2912	vCH_3	100		
2920	vCH_3	93			2917	vCH_3	100		
2921	vCH_3	93			2917	vCH_3	100		

[a] Frequencies in cm^{-1}. Symbols represent; v, stretching; β, bending; w, out-of-plane deformations (wags); t, torsional deformations; r, rocking, δ, methyl group deformations, and tw twisting modes. The potential energy contribution is listed as a percentage in parentheses. Only internal coordinate contributing more than 10% to the potential energy distribution are reported.

Figure S1. Adiabatic torsional potential energy surface of DMA for the (**a**) O_{2A}-As-O_{SA}-C_{T3}, (**b**) O_{SA}-As-O_{SA}-C_{T3} and (**c**) As-O_{SA}-C_{T3}-H_A the black solid line was the HF/6-31G * results. The red dash line was the empirical results which fitted to the HF/6-31G * results.

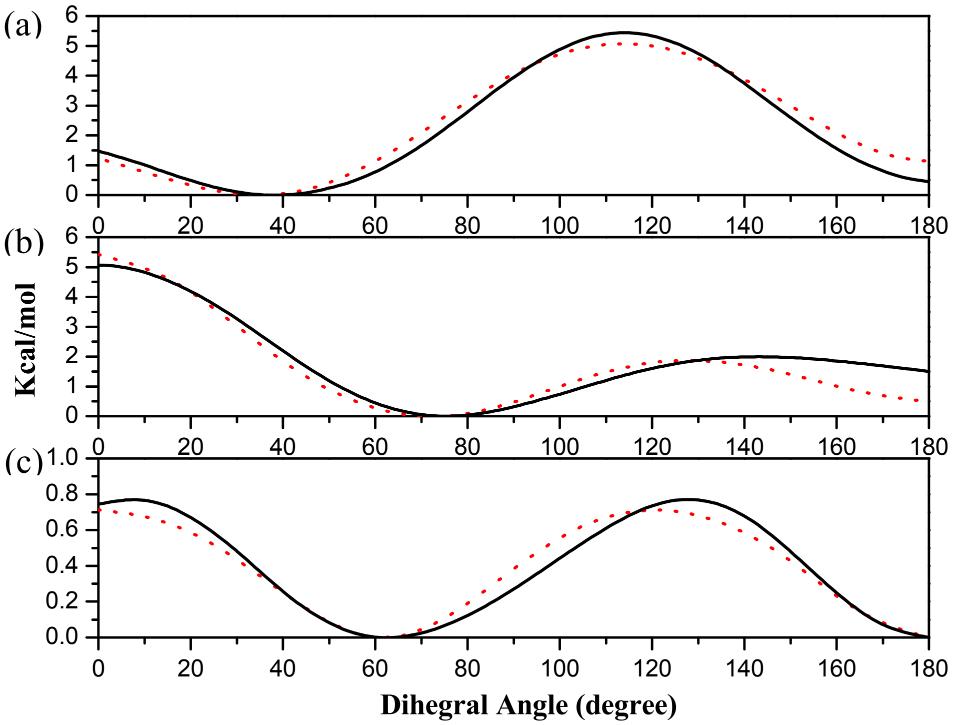

© 2013 by the authors; licensee MDPI, Basel, Switzerland. This article is an open access article distributed under the terms and conditions of the Creative Commons Attribution license (http://creativecommons.org/licenses/by/3.0/).
